# Research Advances in Plant Genomics

**DOI:** 10.3390/life11121313

**Published:** 2021-11-28

**Authors:** Jitendra Kumar, Krishan Mohan Rai

**Affiliations:** 1 Department of Agronomy and Plant Genetics, University of Minnesota, Minneapolis, MN 55108, USA; 2 Department of Microbial and Plant Biology, University of Minnesota, Minneapolis, MN 55108, USA

Breeding efforts have helped in increasing crop yields globally. However, the ever-increasing world population coupled with abiotic and biotic stresses threatens food security for millions of people worldwide. Plant genomics have demonstrated interesting insights into the mechanisms associated with development, and stress responses and can be exploited for improving crop yield.

The review and research articles published in this Special Issue of Life highlight several aspects of advances in plant genomics. For example, Slavokhotova et al. [1] dissected the defense response of an inbred line of *Cucumis sativus* L., which is highly susceptible to cucumber green mottle mosaic virus (CGMMV). The article documents that host silencing machinery was strongly suppressed in infected plants, while the salicylic acid and ethylene signaling pathways were induced. 

Resistant and susceptible lines respond differently to a pathogen attack. The article by Kumar et al. [2] documents the response of a resistant vs. susceptible wheat cultivar in response to wheat dwarf India virus (WDIV) using transcriptome analysis. The differentially expressed genes were found to be involved in signaling, hormone metabolism, enzymes, secondary metabolites, proteolysis, and transcription factors. The study showed that the virus resistance in Sonalika, a resistant cultivar, is likely due to a strong intracellular surveillance mechanism mediated by PR proteins (PR1, RAR1, and RPM1) and ChiB.

Thermo-priming, a short exposure to high temperature, prepares plants for subsequent exposure to high temperature. Kushawaha et al. [3] compared transcriptome and small RNA of rice seedlings after exposing them to high temperatures with and without thermo-priming. The study identified several microRNAs (miRs) such as osa-miR531b, osa-miR5149, osa-miR168a-5p, osa-miR1846d-5p, osa-miR5077, osa-miR156b-3p, osa-miR167e-3p, and their target genes which were heat shock activators or repressors and may serve as important regulatory nodes for plant survival under high temperatures. 

Male sterility is used as a tool by breeders for developing hybrid varieties. The article by Hu et al. [4] investigates the molecular changes during flower development in fertile and sterile cabbage lines. Transcriptome analysis showed high activity for transcription factors belonging to AP2, MYB, bHLH, and WRKY families in the fertile line, and weak transcriptional activity in the sterile line, which may have led to the abnormal stamen development in the sterile line. 

*Fritillaria hupehensis* (Hsiao et K.C. Hsia) is one of the most utilized medicinal plants in China. Guo et al. [5] did a PacBio RS II mediated sequencing using the bulb, leaf, root, and stem tissues of *F. hupehensis*. They evaluated potential metabolic pathways such as flavone and flavonol biosynthesis, vitamin B6 metabolism, valine, leucine, and isoleucine biosynthesis, TGF-beta signaling pathway, ubiquinone and other terpenoid-quinone biosynthesis. They also discovered genomic-SSRs which will help in developing markers for marker-assisted trait-specific breeding in *F. hupehensis*. 

Dendrobium Nestor is an orchid species that is known for its diversity of flower colorations. Cui et al. [6] did transcriptome profiling on petal samples at three developmental stages—flower bud, half bloom, and full bloom—to uncover the molecular mechanism underlying the flower color formation. The study identified genes involved in phenylpropanoid–flavonoid–anthocyanin biosynthesis such as phenylalanine ammonia lyase, chalcone synthase, anthocyanidin synthase, and UDP-flavonoid glucosyl transferase as upregulated in half bloom and full bloom stages. Genes related to auxin, ethylene, cytokinins, salicylic acid, brassinosteroid, and abscisic acid were found to be differentially expressed, and might play important roles in color formation.

CRISPR/Cas has revolutionized genetic engineering in plants. The article by Alok et al. [7] discusses the technical aspect of various important elements that are important for the efficient editing of the desired plant genome. Factors such as promoter regulating the expression of Cas and gRNA, gRNA size, Cas variants, multicistronic gRNA, and vector backbone, influence plant genetic transformation and editing frequency as well. For example, polycistronic tRNA-gRNA and Csy4-gRNA enhance editing efficiency. Similarly, the information on the availability of Cas endonucleases variants, such as Cas9, Cas12a, Cas12b, Casɸ, and CasMINI, with diverse recognition sites has broadened the scope of editing. This review will in fact serve as an encyclopedia for plant-specific CRISPR vectors.

The information generated by genome sequencing, assembly, and annotation projects can help us in developing climate-resilient cultivars. The article by Zenda et al. [8] summarizes recent developments in plant genomics and their application, with an emphasis on cereal crops. They discuss genome sequencing approaches, quantitative trait loci (QTL) mapping and genome-wide association (GWAS) studies, directed mutagenesis, plant non-coding RNAs, gene editing using CRISPR-Cas9, and complementation of crop genotyping by crop phenotyping. 

Noncoding RNAs (ncRNAs) modulate mRNA or protein profiles. ncRNAs are essential for growth and development in plants and have emerged as key players in guarding plants during stress conditions. The smooth functioning of plants under stress is ensured by chloroplasts and mitochondria. These organelles contain crucial metabolic pathways that are required to maintain cell homeostasis. The article by Anand et al. [9] provides detail on ncRNAs origination and their gene regulation in chloroplasts and plant mitochondria.

We hope this Special Issue will provide the reader with an advancement in knowledge in plant genomics, capturing the attention of both specialists and beginners who may be interested in this exciting field.
